# Novel therapeutic strategy for melanoma based on albendazole and the CDK4/6 inhibitor palbociclib

**DOI:** 10.1038/s41598-022-09592-0

**Published:** 2022-04-05

**Authors:** Lin Zhu, Qin Yang, Rong Hu, Yanan Li, Yuanliang Peng, Hong Liu, Mao Ye, Bin Zhang, Peihe Zhang, Feng Liu-Smith, Hui Li, Jing Liu

**Affiliations:** 1 Molecular Biology Research Center, Center for Medical Genetics, Hunan Province Key Laboratory of Basic and Applied Hematology, Medical Genetics and School of Life Sciences, Central South University, Changsha, 410078 China; 2 Department of Dermatology, Hunan Key Laboratory of Skin Cancer and Psoriasis, Hunan Engineering Research Center of Skin Health and Disease, Xiangya Clinical Research Center for Cancer Immunotherapy, Xiangya Hospital, Central South University, Changsha, 410008 Hunan China; 3 School of Medical Laboratory, Shao Yang University, Shaoyang, 422000 Hunan Province China; 4 Molecular Science and Biomedicine Laboratory, State Key Laboratory for Chemo/Biosensing and Chemometrics, College of Biology, College of Chemistry and Chemical Engineering, Collaborative Innovation Center for Chemistry and Molecular Medicine, Hunan University, Changsha, 410082 Hunan China; 5 Department of Histology and Embryology, Xiangya School of Medicine, Central South University, Changsha, 410013 China

**Keywords:** Cancer, Drug discovery, Molecular biology

## Abstract

Although an increasing number of patients benefit from immunotherapy and targeted therapies, melanoma remains incurable with increasing incidence. Drug repositioning and repurposing is an alternative strategy to discover and develop novel anticancer drugs or combined therapeutic regimens. In this study, we demonstrated that albendazole (ABZ), an Food and Drug Administration (FDA)-approved broad-spectrum antiparasitic agent, significantly inhibits the proliferation of melanoma cells in vitro and in vivo. RNA sequencing and flow cytometry analysis revealed that ABZ arrests melanoma cells at the G2/M phase of the cell cycle and induces cell apoptosis. More importantly, the CDK4/6 inhibitor palbociclib, as a member of the first and only class of highly specific CDK inhibitors approved for cancer treatment to date, showed significant synergistic effects with ABZ treatment in melanoma cells and mouse models. Taken together, we revealed a previously unappreciated function of ABZ in antimelanoma proliferation by inducing cell cycle arrest and apoptosis and provided a novel combined therapeutic regimen of ABZ plus CDK4/6 inhibitor treatment in melanoma.

## Introduction

Melanoma is a highly aggressive skin tumor with an increasing incidence and poor prognosis in the metastatic stage^1^. Although targeted therapies and immunotherapies have greatly improved the prognosis of melanoma patients, adaptive resistance and low response rates have resulted in melanoma remaining incurable^2^. Therefore, novel treatment strategies for melanoma are still urgently needed. Drug repositioning and repurposing is an alternative strategy to discover and develop novel treatment schemes^3^.

Albendazole (ABZ) is an FDA-approved broad-spectrum antiparasitic agent against hydatid cysts and neurocysticercosis with low toxicity^4^. In recent years, ABZ has been increasingly recognized as an effective anticancer agent in several cancer cell types. In hepatocellular carcinoma (HCC), ABZ dramatically inhibited the proliferation of all human, rat and mouse HCC cells and arrested SKHEP-1 cells at both the G0/G1 (250 nM) and G2/M (1000 nM) phases of the cell cycle^5^. ABZ also showed anticancer activity in vitro and in xenograft models of ovarian cancer and colorectal cancer^6,7^. Patel et al. showed that treatment of small cell lung cancer and metastatic melanoma cells treated with ABZ renders them more sensitive to radiation in a synergistic fashion^8^. Moreover, a phase I clinical trial was performed and showed that ABZ was well-tolerated, relatively safe and possessed antitumor effects in patients with advanced cancer refractory to conventional treatments^9^. In general, ABZ is recognized as a safe drug with low toxicity and is considered promising as an anticancer drug. However, whether ABZ can be used as a compound or an adjuvant for the treatment of melanoma alone or in combination and the underlying mechanism remains unknown.

Selective cyclin kinase 4/6 (CDK4/6) inhibitors, including palbociclib (Ibrance, PD-0332991; Pfizer), are the first and only class of highly specific CDK inhibitors that have passed the clinical development stage and have been approved for cancer treatment to date. Palbociclib is an orally available, specific small-molecule inhibitor of CDK4/6 that is used in combination with aromatase inhibitors in the therapy of postmenopausal women with metastatic breast cancer positive for the estrogen receptor (ER+) but negative for human epidermal growth factor receptor 2 (HER2−)^10^. Moreover, phase 2 trials have shown palbociclib single-agent activity in mantle-cell lymphoma, breast cancer, liposarcoma, and teratoma with reversible neutropenia as the main toxic effect^11^. CDK4/6 regulates the cellular transition from the G1 to the S phase of the cell cycle. Inhibition of this transition by CDK4/6 inhibitors arrests the progression of the cell cycle in the G1/S phase and blocks cell growth^12^.

In this study, we demonstrated that ABZ inhibited the proliferation of melanoma cells in vitro and in vivo. To provide a better understanding of the precise mechanisms of the antimelanoma effect of ABZ, we performed global transcriptomic profiling of the ABZ-treated and untreated human melanoma A375 cells via RNA sequencing. Our results suggest that ABZ inhibited cell growth by arresting the cell cycle at the G2/M phase. More importantly, we found that combining ABZ and palbociclib synergistically decreased cell viability and tumor growth in mouse models. Overall, we provided potential melanoma therapeutic regimens by treatment with ABZ alone or in combination with CDK4/6 inhibitors.

## Materials and methods

### Cell culture

Human melanoma cells (A375 and SK-MEL-28) and human epidermal cells HaCaT were purchased from the Cell Bank of the Chinese Academy of Science (Shanghai, China). All cells were cultured in DMEM included with 10% fetal bovine serum, 100 U/mL penicillin, 100 μg/mL streptomycin (Gibco, USA) and incubated at 37 °C with 5% CO_2_. ABZ and palbociclib (Selleck, USA) were added to the complete medium at the indicated concentrations and times.

### Methylthiazolyldiphenyl-tetrazolium bromide (MTT) assay

Cells were seeded in triplicate in 96-well plates at a density of 3000 cells per well for 48 h before being subjected to ABZ treatment. Subsequently, MTT (Coolaber, Beijing) was added in each well to obtain a final concentration of 5 mg/mL and further incubated for 3 h. At last, the cells and MTT crystals were dissolved in DMSO (Sigma–Aldrich, Shanghai) and used to determine the absorbance at 570 nm by a microplate reader.

### Plate clone formation assay

Cells were seeded in 6-well plates (1,000 cells per well) in a growth medium and, where applicable, subject to albendazole or/and palbociclib treatment. When colonies reached a considerable size, cells were fixed in 4% paraformaldehyde for 10 min, followed by staining with crystal violet solution (Sigma–Aldrich, Shanghai) for another 15 min. Colonies were counted manually using ImageJ software.

### Flow cytometry assay

For cell cycle analysis, cells were harvested and fixed in 70% ethanol at 4 °C for 24 h. The fixed cells were washed with phosphate-buffered saline (PBS) and then incubated PBS containing 20 μg/mL propidium iodide (PI) and 0.2 mg/mL RNase A at room temperature for 15 min. Flow cytometer FACSDxp AthenaTM and AuroraTM (Cytek, USA) was used to detect the stained cells. Flow Jo 10.0 software was used to further analyze the stained cells.

For apoptosis analysis, cells were collected in an EP tube and washed with PBS. Then, 5 µL annexin V fluorescein isothiocyanate (FITC) was added and followed by a 10 min incubation. The mixture was centrifuged at 100*g* for 5 min and the supernatant was discarded. Annexin V FITC and PI staining solution were added to the precipitated cells and the mixture was incubated on ice for 20 min. Flow cytometer FACSDxp AthenaTM and AuroraTM (Cytek, USA) was used to detect the apoptosis cells. Flow Jo 10.0 software was used to further analyze the apoptosis cells.

### RNA-sequencing assay

mRNA, from total RNA extracted using Trizol reagent, was enriched using magnetic beads with Oligo (dT). Then, the obtained mRNA was fragmented to short length via adding Fragmentation Buffer. mRNA fragments with random primers were needed in the first chain cDNA synthesis. Subsequently, Buffer, dNTPs, RNase H and DNA polymerase I were added for the second chain cDNA synthesis. Purification of PCR products was performed using a kit. EB Buffer was used to eluting the products at the end of the repair and processing of A bases. After adding sequencing connectors, agarose gel electrophoresis was used to recover the target size fragments. The resulting fragments were used as a template for PCR amplification to complete the library preparation. The quality control steps included bioanalyzer RNA chip analysis and qRT-PCR for library quantification. Data were provided by GEO. An Illumina sequencer was used to sequence the constructed library.

### Western blot analysis

Briefly, A375 and SK-MEL-28 cells were treated with 0.625 μM or/and 1.25 μM ABZ for 48 h respectively. Then the total proteins were collected and subjected to 10% SDS–PAGE gel for western blot analysis. For western blot analysis, the following antibody dilutions were used: CDK1 (Santa Cruz, sc-54), Cyclin B1 (Santa Cruz, sc-245), p53 (Santa Cruz, sc-126), p-RB1 (Cell Signaling Technology, 8516), RB1 (Cell Signaling Technology, 9313), Cleaved Caspase-3 (Cell Signaling Technology, 9661), CDK4 (Santa Cruz, sc-238969), Cyclin D (Santa Cruz, sc-8396), phospho Histone H3 (Hunan AiFang biological, Co., Ltd, AF00582) and GAPDH (Santa Cruz, sc-47724).

### Mice tumorigenicity assay

All experiments involving animals were following the guidelines of the Animal Ethics Committee from the Third Xiangya Hospital of Central South University and procedures approved by the committee (SYXK 2020-2019, license no. 2019sydw011). All experimental procedures were conducted in strict accordance with the ARRIVE guidelines. A735 cells (1 × 10^6^) were inoculated subcutaneously into 6-week-old female nude athymic BALB/c nude mice. When the tumor grew to 100 mm^3^, the mice were injected with ABZ (50 mg/kg, dissolved in 100 μL sesame oil, 2 Day i.p., Selleck Chemicals, Cat# S1640), a combination of ABZ and palbociclib (Selleck Chemicals, Cat# S1579) or negative control DMSO respectively. The tumor volume and mice weight were measured every 2 days. The tumor volume was estimated according to the following formula: V = width^2^ × length/2. After 3 weeks, the mice were sacrificed by cervical dislocation, and the tumors were dissected from mice, and tissue samples were fixed with 10% formaldehyde for immunohistochemical staining Ki67 (abcam, ab15580), CDK1 and p-RB1 according to standard protocols. Images were obtained using an Olympus photomicroscope (Olympus, Japan).

### Statistical analysis

SPSS was used to analyze the data, which are expressed as the mean ± standard deviation. Univariate analysis of variance was used to determine that the difference in experimental data between groups was statistically significant, and *p* < 0.05 was considered statistically significant.

## Results

### ABZ inhibited the growth of melanoma cells in vitro and in vivo.

**Figure 1:**
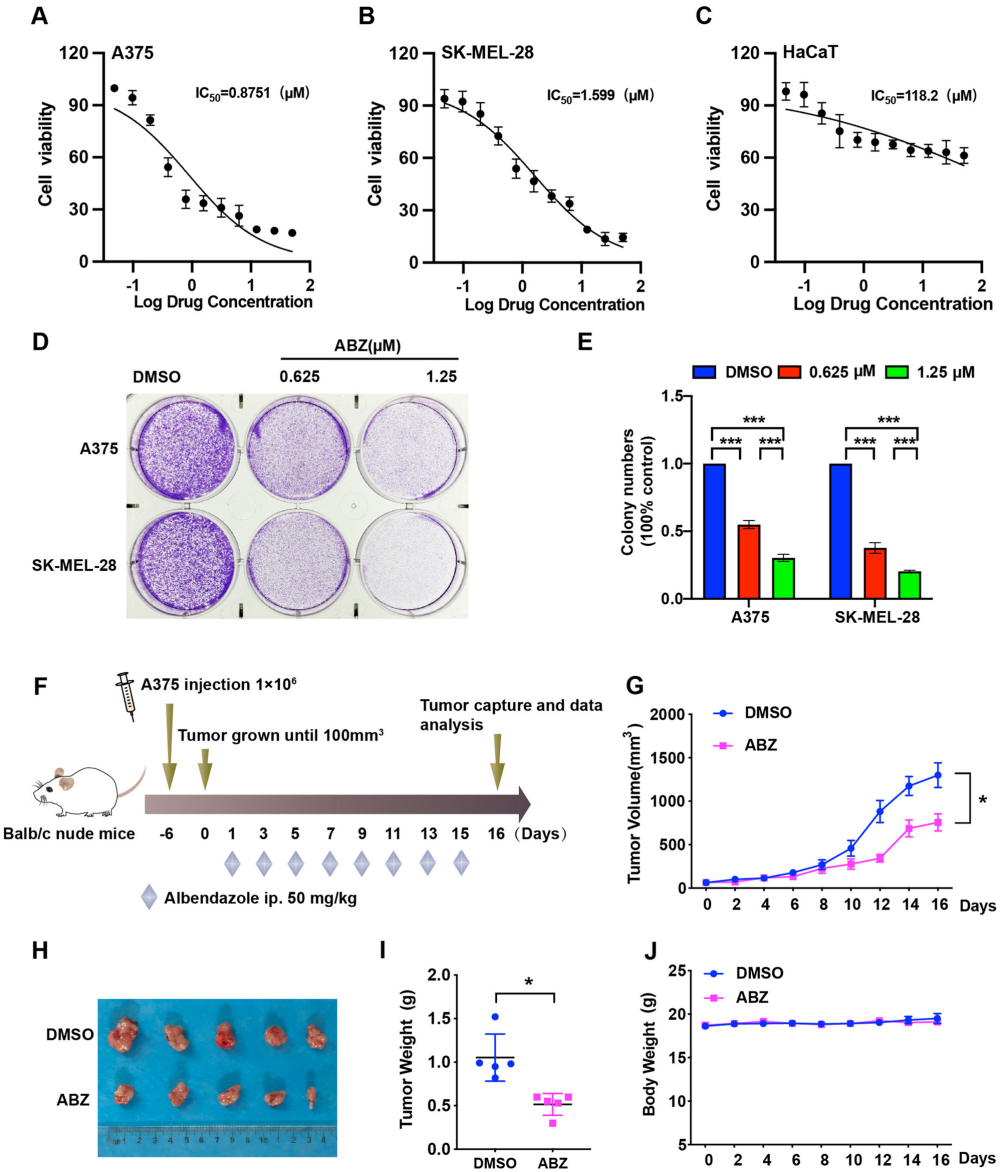
ABZ inhibited melanoma cell growth in vitro and in vivo. A375 (**A**), SK-MEL-28 (**B**) and HaCaT (**C**) cells were treated with various doses of ABZ for 2 days, and MTT assays were performed to determine cell viability. These data were subsequently used to calculate IC50 values. (**D**) Colony growth of A375 and SK-MEL-28 cells treated with the indicated concentrations of ABZ in 6-well plates. (**E**) The number of colonies that formed from A375 and SK-MEL-28 cells was evaluated 14 days after treatment with the indicated concentrations of ABZ. The results are presented as the mean ± SD of three independent experiments, ***p < 0.001. (**F**) A375 cells were injected into mice (n = 5 mice per group) on Day -6, and 50 mg/kg ABZ was administered as indicated. (**G**) Tumor volume was measured at the indicated time points. Data represent mean ± SD, *p < 0.05. (**H**) Representative images of tumors after ABZ treatment at the end of the experiment in the mouse model. (**I**) Tumor weight was measured on Day 16. Data represent mean ± SD, *p < 0.05. (**J**) Mouse weight was measured at the indicated time points.

To investigate the antitumor role of ABZ in melanoma, MTT assays were first performed in melanoma cell lines and human normal keratinocyte HaCaT cells. As shown in Fig. 1A,B, ABZ suppressed the viability of A375 and SK-MEL-28 cells by 50% at very low concentrations, at 0.8751 and 1.599 μM respectively. However, ABZ showed no obvious survival rate change in HaCaT cells, indicating that ABZ exhibits low cytotoxicity in normal cells (Fig. 1C). Colony formation assays also showed that ABZ significantly inhibited melanoma cell proliferation (Fig. 1D,E). To further investigate the antitumor role of ABZ in vivo, we generated subcutaneous melanoma tumor xenografts in nude mice using A375 cells and treated them with ABZ and DMSO (Fig. 1F). As shown in Fig. 1G–I, ABZ treatment significantly reduced the growth and weight of xenografted melanoma tumors compared with DMSO treatment. However, ABZ administration did not result in significant changes in mouse body weight (Fig. 1J), suggesting limited toxicity of ABZ treatment. Taken together, these results suggested that ABZ could significantly inhibit the growth of melanoma cells in vitro and in vivo with low toxicity.

### ABZ arrested the melanoma cells at the G2/M phase and induced cell apoptosis.

**Figure 2:**
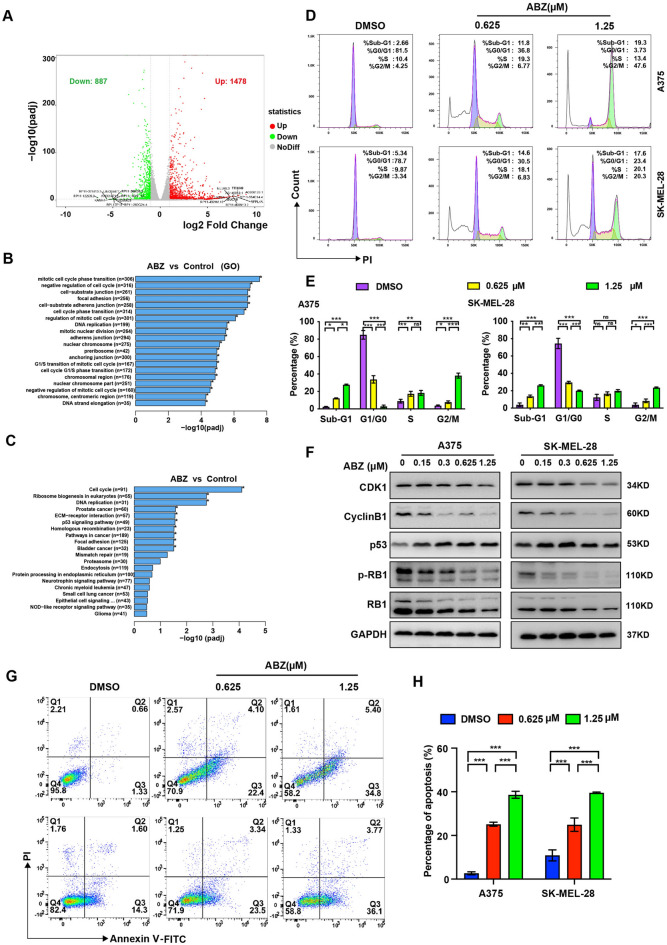
ABZ arrested the melanoma cell cycle at the G2/M phase and induced cell apoptosis. (**A**) Summary of the distribution of differentially expressed genes identified from RNA sequencing data generated from A375 cells with or without ABZ treatment. The average log CPM (count per million) represented the gene expression level. The color represents differentially expressed genes (fold change > 1.5, FDR < 0.05; red: upregulation, green: downregulation, gray: nonsignificant change). B-C. Top enriched GO (**B**) and KEGG (**C**) biological process pathways for genes upregulated or downregulated in A375 cells treated with ABZ. D-E. Representative image (**D**) and quantification (**E**) of flow cytometry analysis of the cell cycle in A375 and SK-MEL-28 cells after treatment with the indicated doses of ABZ. Bar graphs show the quantified results of three independent experiments and are shown as the means ± SD, ^NS^*p* > 0.05, *p < 0.05, **p < 0.01, ***p < 0.001. (**F**) Representative image (full-length gels were presented in Supplementary Figure 1B) of western blot analysis of CDK1, Cyclin B1, p53, p-RB1 and RB1 in A375 and SK-MEL-28 cells after treatment with the indicated doses of ABZ. GAPDH was used as the loading control. Representative image (**G**) and quantification (**H**) of flow cytometry analysis of apoptosis in A375 and SK-MEL-28 cells after treatment with the indicated doses of ABZ. Bar graphs show the quantified results of three independent experiments and are shown as the means ± SD, ***p < 0.001.

To explore the mechanism of action of ABZ in melanoma growth inhibition, we performed RNA-seq analysis to identify the signaling pathways altered by ABZ in A375 cells. We found 1478 significantly upregulated genes and 887 downregulated genes in response to treatment with ABZ (Fig. 2A). In particular, both Gene Ontology (GO) and Kyoto Encyclopedia of Genes and Genomes (KEGG) enrichment analyses revealed that the upregulated genes were significantly enriched in cell cycle-related genes (Fig. 2B,C). To test whether ABZ inhibits melanoma cell growth in a cell cycle-dependent manner, we first performed flow cytometry analysis and showed significant enrichment of cells in the sub-G1 and G2/M phase and a significant reduction in cells in the G0/G1 phase when A375 and SK-MEL-28 cells were treated with ABZ, indicating that ABZ arrested the melanoma cells at the G2/M phase of the cell cycle and induced cell apoptosis (Fig. 2D,E, Supplementary Figure 1A). Furthermore, western blot was performed to analyze cell cycle-associated molecules. As shown in Fig. 2F and Supplementary Figure 1B, the expression levels of CDK1 and Cyclin B1, which are regulatory proteins related to the G2/M phase of the cell cycle, and the level of G1/S phase regulatory protein phospho-RB1 (p-RB1), were significantly reduced in cells treated with ABZ at 0.625 and 1.25 μM compared with DMSO. The expression level of p53, another mediator of the G2/M checkpoint, was increased in cells treated with ABZ. However, the levels of phospho-H3 (p-H3) and γ-H2AX, which are regulatory proteins related to the chromosome condensation during mitosis and DNA damage respectively, were not obviously changed unless at the very high concentration of ABZ (Supplementary Figure 2). In particular, to investigate the apoptosis-inducing effect of ABZ, we analyzed the percentage of apoptotic A375 and SK-MEL-28 cells using flow cytometry. As shown in Fig. 2G,H, the percentage of apoptotic melanoma cells was significantly increased after treatment with the indicated doses of ABZ. Moreover, ABZ treatment significantly increased the Cleaved Caspase-3 in A375 and SK-MEL-28 cells (Supplementary Figure 1B). Taken together, we found that ABZ inhibited the growth of melanoma cells by inducing G2/M phase arrest and apoptosis.

### ABZ plus palbociclib synergistically suppresses cell growth in vitro

**Figure 3:**
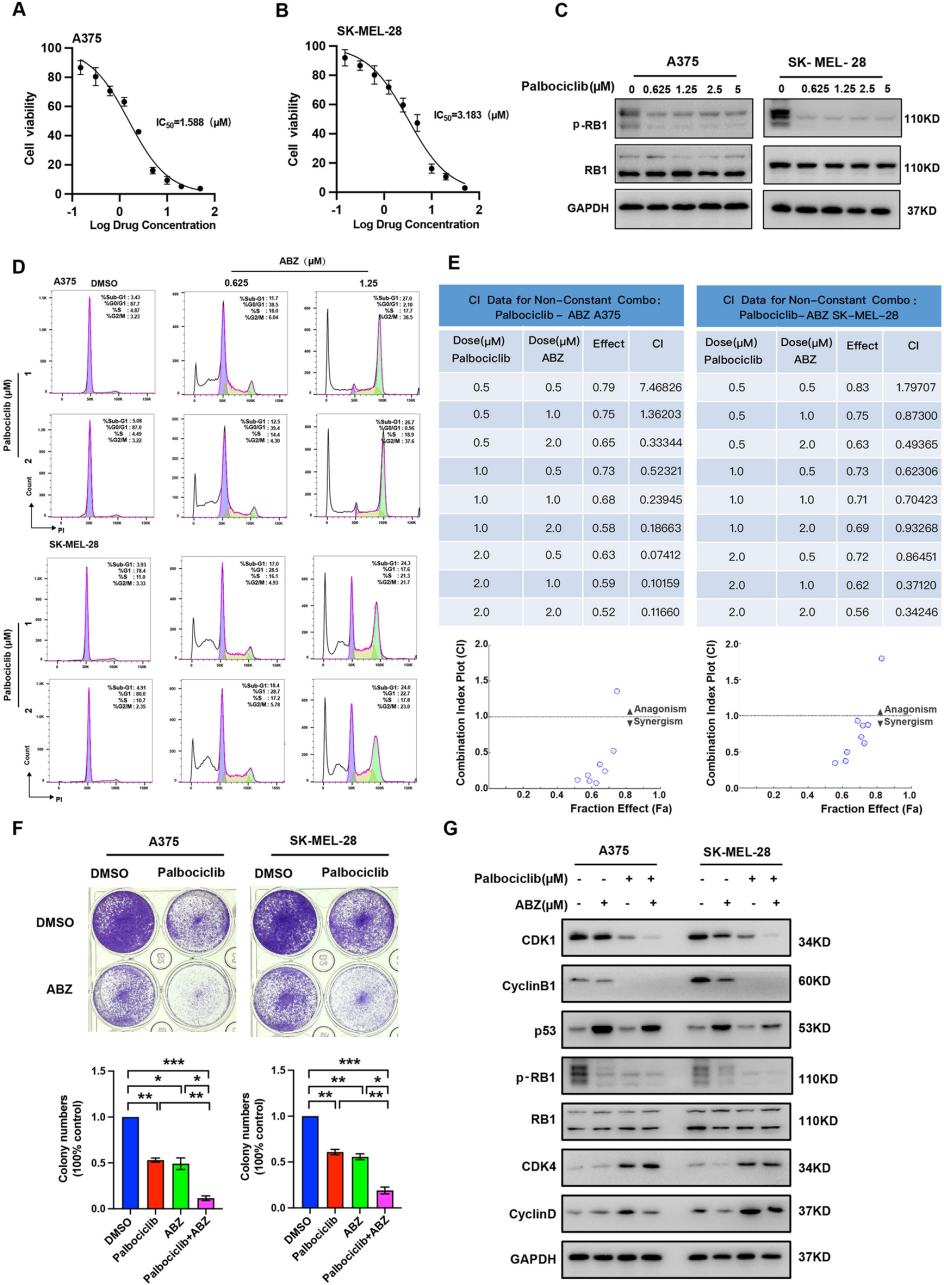
ABZ plus palbociclib synergistically suppresses cell growth in vitro*.* A375 (**A**) and SK-MEL-28 (**B**) cells were treated with various doses of palbociclib for 3 days, and an MTT assay was performed to determine cell survival. These data were used to calculate IC50 values. (**C**) Representative image (full-length gels were presented in Supplementary Figure 2) of western blot analysis of p-RB1 and RB1 in A375 and SK-MEL-28 cells after treatment with the indicated doses of palbociclib. GAPDH was used as the loading control. (**D**) Representative image of flow cytometry analysis of the cell cycle in A375 and SK-MEL-28 cells after co-treatment with the indicated doses of ABZ and palbociclib. (**E**) A375 and SK-MEL-28 cells were treated with different concentrations of ABZ, palbociclib, or ABZ plus palbociclib for 2 days, and cell viability was assessed using the MTT assay. Combination index (CI) values were analyzed using CompuSyn software for a nonconstant drug ratio, where CI < 1 indicates synergism. (**F**) The number of colonies that formed from A375 and SK-MEL-28 cells was evaluated 12 days after treatment with ABZ (0.5 μM), palbociclib (1 μM), or ABZ (0.5 μM) plus palbociclib (1 μM). The results are presented as the mean ± SD of three independent experiments, *p < 0.05, **p < 0.01,***p < 0.001. (**G**) Representative image (full-length gels were presented in Supplementary Figure 3D) of western blot analysis of CDK1, Cyclin B1, p53, p-RB1, RB1, CDK4 and Cyclin D in A375 and SK-MEL-28 cells after treatment with the indicated doses of palbociclib. GAPDH was used as the loading control.

As mentioned above, ABZ inhibited the growth of melanoma cells by inducing G2/M phase arrest. Therefore, we tried to explore whether ABZ exhibits synergistic effects with the cell cycle inhibitor palbociclib, an FDA-approved specific small-molecule inhibitor of CDK4/6, or Vemurafenib (PLX4032), an FDA-approved BRAF kinase inhibitor (BRAFi) for melanoma patients harboring the constitutively active BRAF-V600E mutation. First, we investigated the inhibitory effects of palbociclib on A375 and SK-MEL-28 cell survival based on MTT assays. As shown in Fig. 3A,B, the cell survival ratio was significantly decreased as the concentration of palbociclib increased, and 50% inhibition after 48 h of treatment was observed at palbociclib concentrations of 1.588 and 3.183 μM in A375 (IC50 = 1.588 μM) and SK-MEL-28 (IC50 = 3.183 μM) cells, respectively. Western blot showed that treatment with palbociclib dramatically reduced the level of p-RB1, indicating that palbociclib destructed the G1/S phase of the cell cycle (Fig. 3C, Supplementary Figure 3A). Flow cytometry analysis showed that co-treated the A375 and SK-MEL-28 cells with ABZ and palbociclib significant enrichment of cells in the sub-G1 and G2/M phase and a significant reduction in cells in the G0/G1 phase (Fig. 3D). Next, we combined palbociclib and ABZ at the indicated concentrations to treat A375 and SK-MEL-28 cells and used CompuSyn software to calculate the combination index. As expected, palbociclib and ABZ produced synergistic effects at very low concentrations (both < 1 μM, Fig. 3E, Supplementary Figure 3B). Meanwhile, combining palbociclib and ABZ decreased the colony number more significantly than either treatment alone in A375 and SK-MEL-28 cells (Fig. 3F). Co-treated the A375 and SK-MEL-28 cells with ABZ and palbociclib synergistically reduced the protein level of CDK1, Cyclin B1 and p-RB1, while increasing the accumulation of p53, CDK4 and Cyclin D (Fig. 3G, Supplementary Figure 3C). However, combining ABZ and Vemurafenib (PLX4032) have no synergistic effect on the inhibition of cell growth (Supplementary Figure 4).

### Synergistic effect of ABZ and palbociclib in the treatment of a melanoma xenograft mouse model

**Figure 4:**
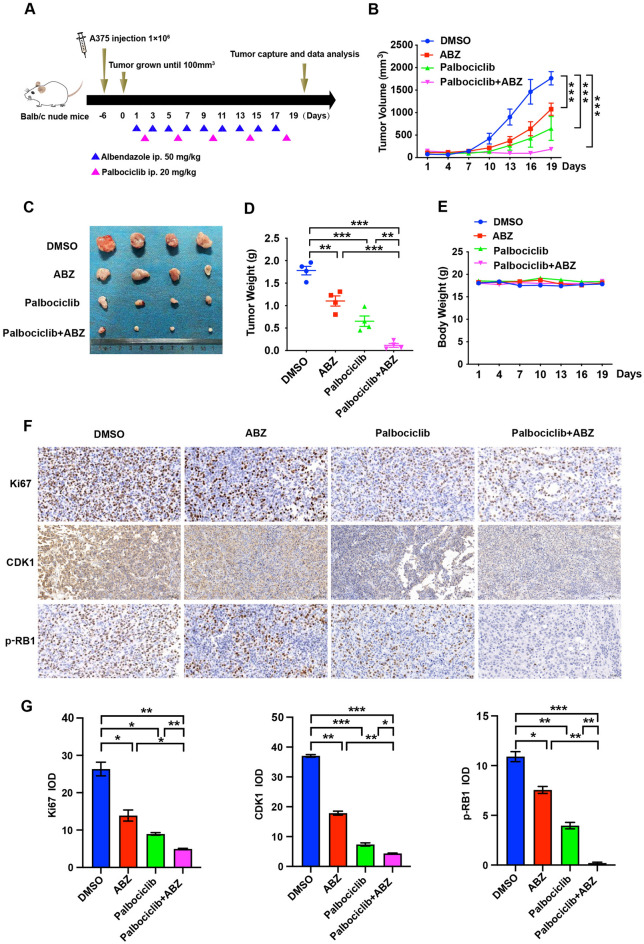
Synergistic effect of ABZ and palbociclib in the treatment of a melanoma xenograft mouse model. (**A**) A375 cells were injected into mice (n = 4 mice per group) on Day -6, and 50 mg/kg ABZ and 20 mg/kg palbociclib were administered as indicated. (**B**) Tumor volume was measured after ABZ, palbociclib, or ABZ plus palbociclib treatment at the indicated time points. Data represent mean ± SD, ***p < 0.001. (**C**) Representative images of tumors after ABZ, palbociclib, or ABZ plus palbociclib treatment at the end of the experiment in the mouse model. (**D**) Tumor weight was measured on Day 19. Data represent mean ± SD, **p < 0.01, ***p < 0.001. (**E**) Mouse weight was measured at the indicated time points. F-G. Representative images (**F**) and quantification (**G**) of immunohistochemistry for Ki67, CDK1 and p-RB1 in tumor sections from mouse xenografted melanoma tumors. Data represent mean ± SD, *p < 0.05, **p < 0.01, ***p < 0.001.

Based on the aforementioned results that ABZ plus palbociclib synergistically suppresses cell growth in vitro, we hypothesized that ABZ plus palbociclib may present a similar synergistic effect in the mouse model. To test this hypothesis, subcutaneous melanoma tumor xenografts in nude mice with A375 cells were generated and treated with ABZ, palbociclib, ABZ plus palbociclib, or the control (Fig. 4A). As shown in Fig. 4B, both ABZ or palbociclib alone significantly decreased tumor growth beginning on Day 13 after treatment compared to the control group, whereas ABZ and palbociclib combination treatment achieved better efficacy. Similarly, combining ABZ and palbociclib reduced tumor volume and tumor weight more significantly than either treatment alone (Fig. 4C,D). More importantly, regardless of whether the mice were treated with ABZ or palbociclib alone or together, no significant changes in mouse weight were noted (Fig. 4E). In addition, ABZ and palbociclib combination treatment markedly reduced the number of proliferative cells in tumors, as indicated by decreased Ki67-positive cells compared to the tumors treated with ABZ or palbociclib alone or control tumors. Furthermore, combining ABZ and palbociclib also dramatically decreased the expression levels of CDK1 and p-RB1 in tumors compared to those treated with ABZ or palbociclib alone or control (Fig. 4F,G). Overall, we suggest that ABZ plus palbociclib synergistically suppresses tumor growth in a melanoma xenograft mouse model with no side effects.

## Discussion

**Figure 5:**
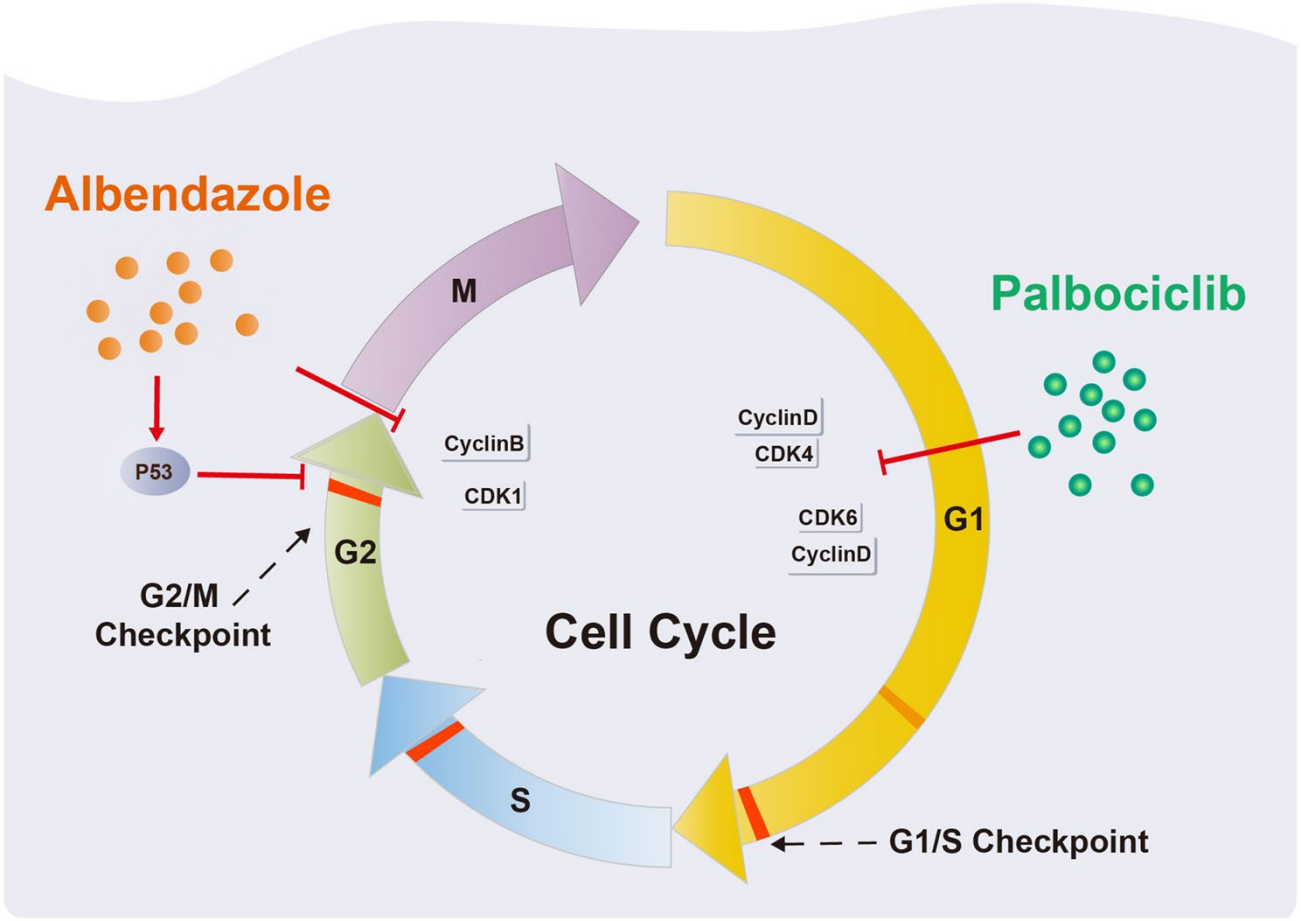
Schematic presentation of the Albendazole plus palbociclib synergistically suppresses melanoma cell growth by arresting the cell cycle.

Melanoma is highly aggressive with an increasing incidence, and the development of novel treatment strategies remains an urgent need^13^. In this study, we demonstrated that ABZ, an FDA-approved broad-spectrum anthelmintic drug, significantly inhibits the proliferation of melanoma cells in vitro and in vivo. Mechanistically, using RNA sequencing and flow cytometry analysis, we revealed that ABZ arrested the melanoma cell cycle at the G2/M phase and induced cell apoptosis. Preclinically, we showed that ABZ had a synergistic tumor-eliminating effect with the CDK4/6 inhibitor palbociclib in the treatment of nude mouse models (Fig. 5). Taken together, we revealed a previously unappreciated function of ABZ in antimelanoma proliferation by inducing cell cycle arrest and apoptosis and provided a novel combined therapeutic regimen of ABZ plus CDK4/6 inhibitor treatment in melanoma.

In recent years, the clinical practice of new drug compounds, especially targeted therapies (i.e., BRAFis) and immune checkpoint inhibitors (ICIs), has largely improved the response rates and overall survival (OS) of advanced melanoma patients^14^. *BRAF* is an oncogene encoding a serine-threonine protein kinase that is mutated in approximately 50% of melanomas^15^. The most promising targeted therapies include the BRAF inhibitors vemurafenib and dabrafenib, which were approved for the treatment of metastatic and unresectable BRAF-mutated melanomas in 2011 and 2013, respectively^16,17^. However, a majority of patients develop secondary resistance to BRAFis within a relatively short amount of time^18^. The combination of BRAFis and MEK inhibitors (MEKi) has recently demonstrated prolonged survival in patients with advanced melanoma harboring activating BRAF mutations. This therapy strategy has demonstrated high anti-tumor activity with fast responses in most patients. However, tumor relapse commonly occurs 12–18 months after initiation of treatment due to the emergence of acquired resistance^19^. We investigated the combined inhibitory effect of the BRAFi vemurafenib and ABZ on wild-type A375 cells and vemurafenib-resistant A375 cells (A375R). Although ABZ could effectively inhibit the growth of vemurafenib-wildtype and -resistant melanoma cells, ABZ plus vemurafenib have no synergistic effect on the inhibition of cell growth, and the underlying mechanism needs to be further explored in our future work. In the presence of BRAF mutations, both BRAFi-targeted therapies and ICIs can be used; however, in the presence of wild-type BRAF, only ICIs can be prescribed^20^. Regarding ICIs, first-line immunotherapy is represented by anti-PD-1 blockade (nivolumab and pembrolizumab), while anti-CTLA-4 ipilimumab can be used as second-line therapy^21^. Although ICIs represent a breakthrough for melanoma therapeutics, a significant subset of patients does not respond to these drugs, and many patients who do respond develop secondary resistance^22,23^.

Considering the toxicity (largely due to off-target effects) and rapid development of adaptive resistance to targeted therapies in addition to the low response ratio and the enormous cost of immunotherapies, it is important to identify economical and effective existing drugs. Herein, we believe that ABZ is such a compound that exhibits great potential for future clinical use in melanoma therapy. We showed that ABZ inhibits melanoma cell proliferation in vitro with very low IC50 values of approximately 1000 nM. Moreover, ABZ significantly suppressed the growth of xenografted melanoma tumors with low toxicity. These results indicated that ABZ is an existing drug that may serve as a potentially effective melanoma therapy. However, whether ABZ can be used as an adjuvant for the treatment of melanoma in combination with other drugs still needs to be further explored.

Cyclin-dependent kinases (CDKs) are a family of serine/threonine kinases that regulate cell cycle progression through the G1, S, G2 and M phases, and aberrant expression or mutations in CDKs consistently result in cell cycle dysregulation and cancer^24^. Therefore, CDKs have been recognized as promising therapeutic targets, fueling efforts in the development of CDK inhibitors as anticancer drugs^25^. Palbociclib, a selective CDK4/6 inhibitor, was approved for the treatment of postmenopausal women with metastatic breast cancer^26^. Because ABZ inhibited the proliferation of melanoma cells by arresting the cell cycle at the G2/M phase, we further assessed whether ABZ combined with palbociclib, which arrested the cell cycle in the G1/S phase, could synergistically suppress cell growth. Our data showed that ABZ plus palbociclib synergistically suppresses cell growth in vitro at very low concentrations (500–1000 nM). More importantly, combination treatment with ABZ significantly enhanced the tumor elimination effect of palbociclib in mouse models. However, the molecular mechanism underlying the combination treatment needs more in-depth work to elucidate in the future.

Taken together, our results suggest that ABZ inhibits the proliferation of melanoma cells by inducing cell cycle arrest and apoptosis, and combination treatment with ABZ and a CDK4/6 inhibitor exhibits potential as a novel therapeutic strategy for the treatment of melanoma.

## Supplementary Information

Supplementary Figures.

## References

[1] Siegel RL, Miller KD, Fuchs HE, Jemal A (2021). Cancer statistics, 2021. CA Cancer J. Clin..

[2] Kakavand H, Wilmott JS, Long GV, Scolyer RA (2016). Targeted therapies and immune checkpoint inhibitors in the treatment of metastatic melanoma patients: A guide and update for pathologists. Pathology.

[3] Antoszczak M, Markowska A, Markowska J, Huczynski A (2020). Old wine in new bottles: Drug repurposing in oncology. Eur. J. Pharmacol..

[4] Gil-Grande LA, Rodriguez-Caabeiro F, Prieto JG, Sanchez-Ruano JJ, Brasa C, Aguilar L, Garcia-Hoz F, Casado N, Barcena R, Alvarez AI, Dal-Re R (1993). Randomised controlled trial of efficacy of albendazole in intra-abdominal hydatid disease. Lancet.

[5] Pourgholami MH, Woon L, Almajd R, Akhter J, Bowery P, Morris DL (2001). In vitro and in vivo suppression of growth of hepatocellular carcinoma cells by albendazole. Cancer Lett..

[6] Noorani L, Stenzel M, Liang R, Pourgholami MH, Morris DL (2015). Albumin nanoparticles increase the anticancer efficacy of albendazole in ovarian cancer xenograft model. J. Nanobiotechnol..

[7] Pourgholami MH, Akhter J, Wang L, Lu Y, Morris DL (2005). Antitumor activity of albendazole against the human colorectal cancer cell line HT-29: In vitro and in a xenograft model of peritoneal carcinomatosis. Cancer Chemother. Pharmacol..

[8] Patel K, Doudican NA, Schiff PB, Orlow SJ (2011). Albendazole sensitizes cancer cells to ionizing radiation. Radiat. Oncol..

[9] Pourgholami MH, Szwajcer M, Chin M, Liauw W, Seef J, Galettis P, Morris DL, Links M (2010). Phase I clinical trial to determine maximum tolerated dose of oral albendazole in patients with advanced cancer. Cancer Chemother. Pharmacol..

[10] Toogood PL, Harvey PJ, Repine JT, Sheehan DJ, VanderWel SN, Zhou H, Keller PR, McNamara DJ, Sherry D, Zhu T, Brodfuehrer J, Choi C, Barvian MR, Fry DW (2005). Discovery of a potent and selective inhibitor of cyclin-dependent kinase 4/6. J. Med. Chem..

[11] Clark AS, Karasic TB, DeMichele A, Vaughn DJ, O'Hara M, Perini R, Zhang P, Lal P, Feldman M, Gallagher M, O'Dwyer PJ (2016). Palbociclib (PD0332991)—A selective and potent Cyclin-Dependent kinase inhibitor: A review of pharmacodynamics and clinical development. JAMA Oncol..

[12] Choi YJ, Anders L (2014). Signaling through cyclin D-dependent kinases. Oncogene.

[13] Davis LE, Shalin SC, Tackett AJ (2019). Current state of melanoma diagnosis and treatment. Cancer Biol. Ther..

[14] Korn EL, Liu PY, Lee SJ, Chapman JA, Niedzwiecki D, Suman VJ, Moon J, Sondak VK, Atkins MB, Eisenhauer EA, Parulekar W, Markovic SN, Saxman S, Kirkwood JM (2008). Meta-analysis of phase II cooperative group trials in metastatic stage IV melanoma to determine progression-free and overall survival benchmarks for future phase II trials. J Clin. Oncol..

[15] Akbani R, Akdemir KC, Aksoy BA, Albert M, Ally A, Amin SB, Arachchi H, Arora A, Auman JT, Ayala B, Baboud J (2015). Genomic classification of cutaneous melanoma. Cell.

[16] Rebecca VW, Sondak VK, Smalley KS (2012). A brief history of melanoma: From mummies to mutations. Melanoma Res..

[17] Chapman PB, Hauschild A, Robert C, Haanen JB, Ascierto P, Larkin J, Dummer R, Garbe C, Testori A, Maio M, Hogg D, Lorigan P, Lebbe C, Jouary T, Schadendorf D, Ribas A, O'Day SJ, Sosman JA, Kirkwood JM, Eggermont AM, Dreno B, Nolop K, Li J, Nelson B, Hou J, Lee RJ, Flaherty KT, McArthur GA (2011). Improved survival with vemurafenib in melanoma with BRAF V600E mutation. N. Engl. J. Med..

[18] Chan MM, Haydu LE, Menzies AM, Azer MW, Klein O, Lyle M, Clements A, Guminski A, Kefford RF, Long GV (2014). The nature and management of metastatic melanoma after progression on BRAF inhibitors: Effects of extended BRAF inhibition. Cancer Am. Cancer Soc..

[19] Specenier P (2020). An overview of binimetinib for the treatment of melanoma. Expert Opin. Pharmacother..

[20] Keilholz U, Ascierto PA, Dummer R, Robert C, Lorigan P, van Akkooi A, Arance A, Blank CU, Chiarion SV, Donia M, Faries MB, Gaudy-Marqueste C, Gogas H, Grob JJ, Guckenberger M, Haanen J, Hayes AJ, Hoeller C, Lebbe C, Lugowska I, Mandala M, Marquez-Rodas I, Nathan P, Neyns B, Olofsson BR, Puig S, Rutkowski P, Schilling B, Sondak VK, Tawbi H, Testori A, Michielin O (2020). ESMO consensus conference recommendations on the management of metastatic melanoma: Under the auspices of the ESMO Guidelines Committee. Ann. Oncol..

[21] Quaglino P, Fava P, Tonella L, Rubatto M, Ribero S, Fierro MT (2021). Treatment of advanced metastatic melanoma. Dermatol. Pract. Concept.

[22] Rodriguez-Cerdeira C, Carnero GM, Lopez-Barcenas A, Sanchez-Blanco E, Sanchez-Blanco B, Fabbrocini G, Bardhi B, Sinani A, Guzman RA (2017). Advances in immunotherapy for melanoma: A comprehensive review. Mediat. Inflamm..

[23] Albittar AA, Alhalabi O, Glitza OI (2020). Immunotherapy for melanoma. Adv. Exp. Med. Biol..

[24] Asghar U, Witkiewicz AK, Turner NC, Knudsen ES (2015). The history and future of targeting cyclin-dependent kinases in cancer therapy. Nat. Rev. Drug Discov..

[25] Otto T, Sicinski P (2017). Cell cycle proteins as promising targets in cancer therapy. Nat. Rev. Cancer.

[26] Bellet M, Ahmad F, Villanueva R, Valdivia C, Palomino-Doza J, Ruiz A, Gonzalez X, Adrover E, Azaro A, Valls-Margarit M, Parra JL, Aguilar J, Vidal M, Martin A, Gavila J, Escriva-de-Romani S, Perello A, Hernando C, Lahuerta A, Zamora P, Reyes V, Alcalde M, Masanas H, Celiz P, Ruiz I, Gil M, Segui MA, de la Pena L (2019). Palbociclib and ribociclib in breast cancer: Consensus workshop on the management of concomitant medication. Ther. Adv. Med. Oncol..

