# Supplementary material for: Novel therapeutic strategy for melanoma based on albendazole and the CDK4/6 inhibitor palbociclib
Source: Sci Rep. 2022 Apr 5;12:5706. doi: 10.1038/s41598-022-09592-0 (PMC8983746; doi:10.1038/s41598-022-09592-0)
Supplement: Supplementary file 1 — Supplementary Figures. [file 41598_2022_9592_MOESM1_ESM.docx]

**Supplementary Figure 1**

**
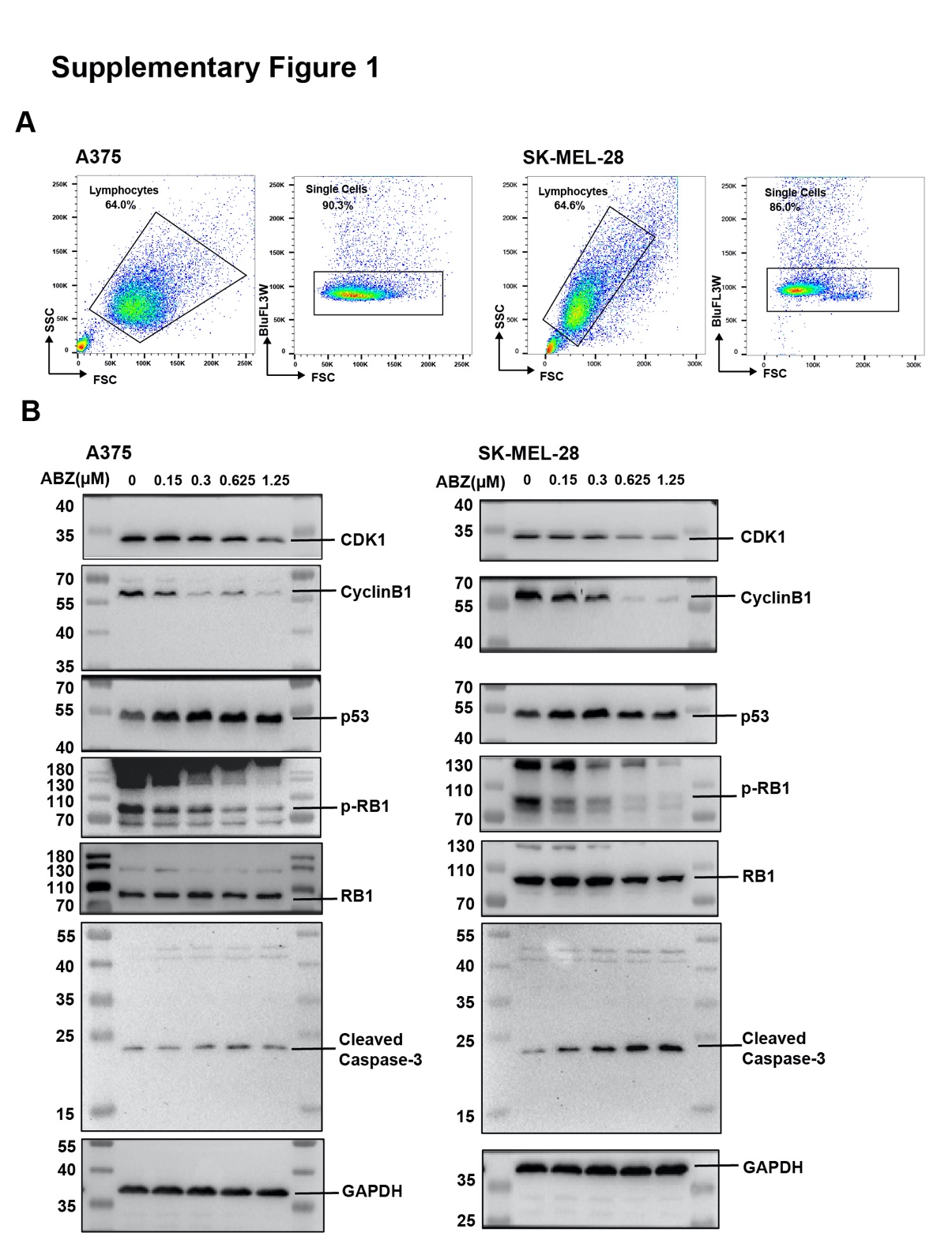
**

Supplementary Figure 1. A. The gating process of FACS experiments for cell cycle in A375 and SK-MEL-28 cells. B. Images of blots with membrane edges and protein markers for Figure 2F.

**Supplementary Figure 2**


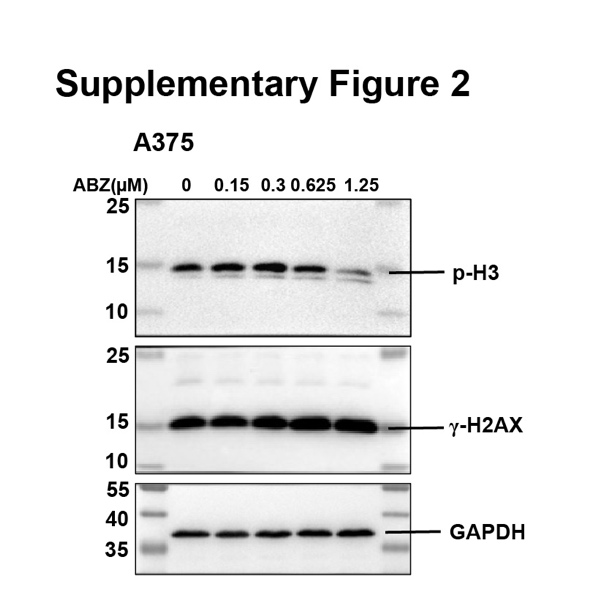


Supplementary Figure 2. Representative image (images of blots with membrane edges and protein markers) of western blot analysis of p-H3 and γ-H2AX in A375 cells after treatment with the indicated doses of ABZ. GAPDH was used as the loading control.

**Supplementary Figure 3**


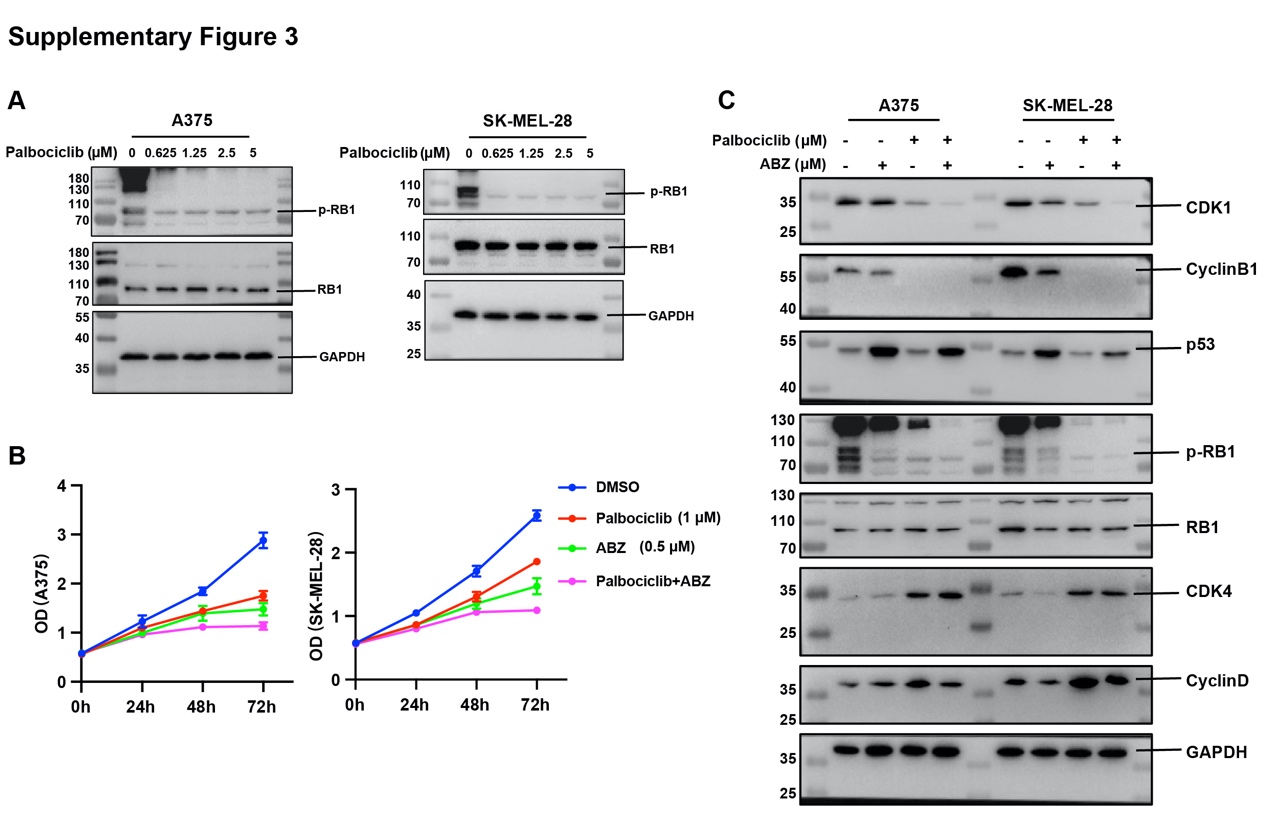


Supplementary Figure 3. A. Representative image (images of blots with membrane edges and protein markers) for Figure 3C of western blot analysis of p-RB1 and RB1 in A375 and SK-MEL-28 cells after treatment with the indicated doses of palbociclib. GAPDH was used as the loading control. B-C. The growth curves for A375 (B) and SK-MEL-28 (C) cells treated with representative synergistic effect doses of ABZ (0.5 μM) and palbociclib (1 μM) at the indicated time points. D. Images of blots with membrane edges and protein markers for Figure 3G.

**Supplementary Figure 4**


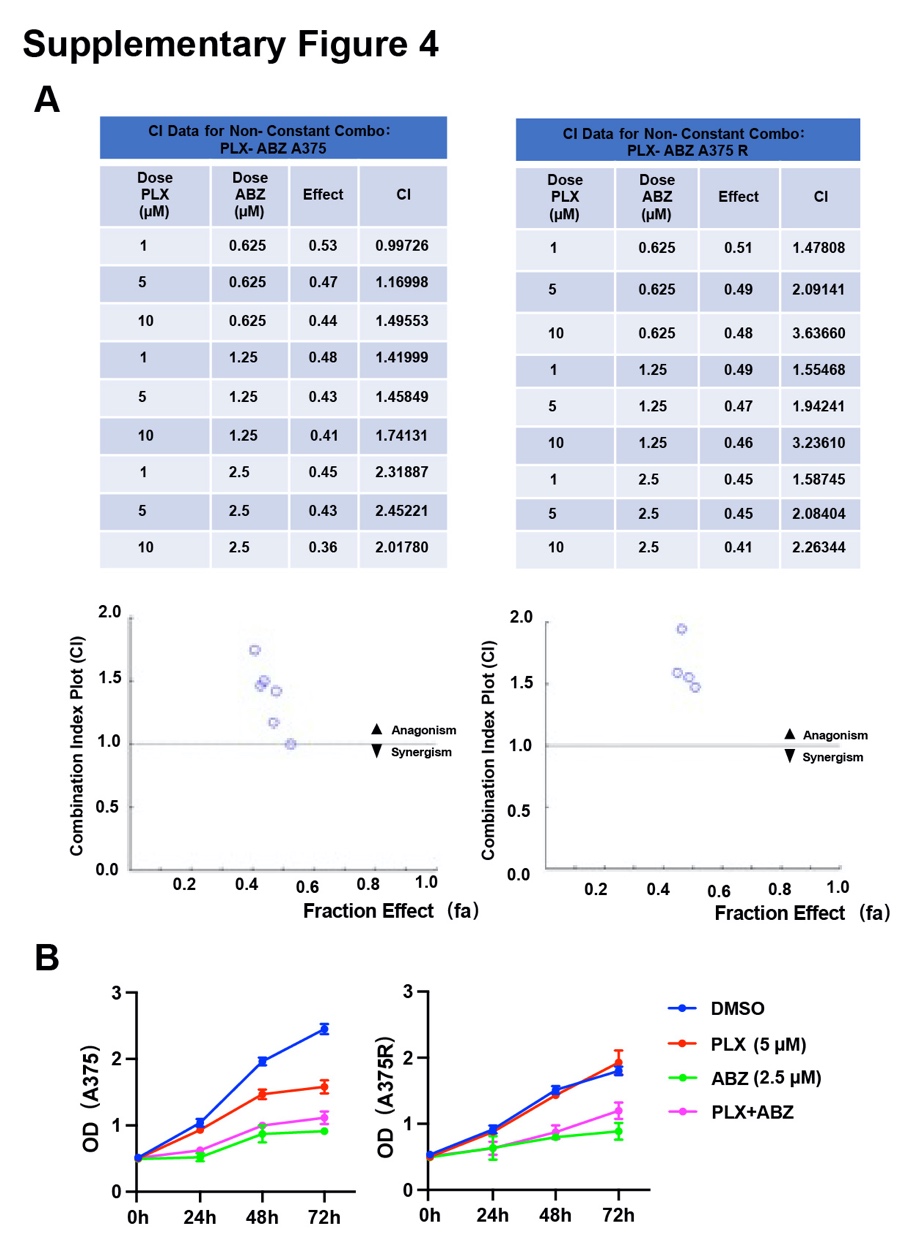


Supplementary Figure 4. A. A375 and SK-MEL-28 cells were treated with different concentrations of ABZ, Vemurafenib, or ABZ plus Vemurafenib for 2 days, and cell viability was assessed using the MTT assay. Combination index (CI) values were analyzed using CompuSyn software for a nonconstant drug ratio, where CI<1 indicates synergism. B. The growth curves for A375 and SK-MEL-28 cells treated with representative synergistic effect doses of ABZ (2.5 μM) and Vemurafenib (5 μM) at the indicated time points.
